# Breast Milk Jaundice and Maternal Diet with Chinese Herbal Medicines

**DOI:** 10.1155/2012/150120

**Published:** 2012-07-03

**Authors:** Yi-Hao Weng, Ya-Wen Chiu, Shao-Wen Cheng

**Affiliations:** ^1^Department of Pediatrics, Chang Gung Memorial Hospital at Taipei, Chang Gung University College of Medicine, Taipei 105, Taiwan; ^2^Division of Preventive Medicine and Health Services Research, Institute of Population Health Sciences, National Health Research Institutes, Miaoli 350, Taiwan; ^3^School of Public Health, College of Public Health and Nutrition, Taipei Medical University, Taipei 110, Taiwan

## Abstract

Our objective was to identify the association between maternal diet with Chinese herbal medicines and prolonged jaundice of breast-fed infants. Healthy infants at 25 to 45 days of age were eligible for enrollment into this prospective study. Jaundice was defined as a transcutaneous bilirubin (TcB) value ≥ 5 mg/dL. A questionnaire survey asking feeding type, stool pattern, and maternal diet was conducted at the time of TcB measurement. A total of 1148 infants were enrolled, including 151 formula-fed, 436 combination-fed, and 561 breast-fed infants. The incidences of jaundice were 4.0% in formula-fed infants, 15.1% in combination-fed infants, and 39.8% in breast-fed infants (*P* < 0.001). In addition, jaundice was noted in 37.1% of preterm infants and 25.0% of term infants (*P* < 0.001). Furthermore, jaundice was more common in breast-fed infants whose mothers did not consume the traditional Chinese herbal medicines than in breast-fed infants whose mothers did consume such medicines (*P* < 0.001). In conclusion, this cohort study has identified late-preterm birth and breast feeding as the contributory factors for prolonged jaundice of apparently well infants. The data indicate that postpartum diet with Chinese herbal medicines is associated with breast milk jaundice.

## 1. Introduction

Jaundice is the most commonly evaluated condition of well infants. It is associated with a variety of physiologic and pathologic conditions [1]. Prolonged jaundice, defined as visible jaundice beyond 14 days, can be a sign of a serious underlying pathology [2]. Nevertheless, the vast majority of prolonged jaundice cases are of benign origin. Many studies have documented the strong association between the feeding of breast milk and an increase in the risk of prolonged jaundice [3, 4]. The mechanism of breast milk jaundice is not clearly understood yet. A number of theories have arisen to explain it, including environmental and genetic factors [5–9]. To date, a correlation between maternal diet and breast milk jaundice has not been verified. Intake of Chinese herbs has been proposed as a useful treatment for neonatal hyperbilirubinemia [10–12]. However, the impact of maternal consumption with Chinese herbal medicines on breast milk jaundice is not clear.

This prospective cohort survey was conducted to determine the correlation of maternal diet with prolonged jaundice among healthy infants at one month of age. This study will shed some light on the management of prolonged jaundice for apparently well infants.

## 2. Materials and Methods

### 2.1. Study Subjects

Healthy infants at 25 to 45 days of age were eligible for enrollment at the outpatient clinic of the Chang Gung Memorial Hospital at Taipei between December 2010 and November 2011. Those with gestational age less than 34 weeks, birth weight less than 2000 grams, or illness were excluded. The Institutional Review Board of Chang Gung Memorial Hospital approved the study protocol.

### 2.2. Study Design

Each infant had a transcutaneous bilirubin (TcB) measurement using a portable BiliCheck device (Spectrx Inc, Norcross, GA). The BiliCheck system averaged the spectra of five replicate measurements on the forehead to give a bilirubin estimate. Concurrent body weight was measured to investigate the rate of weight gain. The devices for measuring the weight and TcB value were the same through the whole study period.

The parents or guardians of enrolled infants were surveyed. Three questions were asked.

- *Feeding Type*. The feeding type was classified into three categories: (1) formula feeding; (2) combination feeding of breast milk and formula, defined as at least one meal of breast milk and formula daily; (3) breast milk feeding.
- *Stool Pattern*. The stool pattern was determined by the frequency of stool output, classified into three categories: (1) more than four times per day; (2) two to four times per day; (3) fewer than two times per day.
- *Maternal Diet*. Whether the mother routinely consumed a traditional Chinese diet during the postpartum period was asked. The traditional Chinese diet for new mothers was defined as an intake of Chinese herbal medicines (including *sh*ē*ng-huà tāng*, *sì wù tāng* and *Eucommia ulmoides*) along with so-called warm food (such as chicken soup and sesame-oil chicken) [13]. This diet is part of a traditional month-long period of customs aimed at accelerating the recovery of postpartum mothers [14]. The other Chinese herbal medicines were not examined in this study.

### 2.3. Collection of Demographic Data

Demographic data—including gender, birth weight, and gestational age—were collected from birth records. All infants were screened for G6PD deficiency on the third day of life with blood samples from heel stick by hemiquantitative fluorescent spot test, which is a routine part of Taiwan's national newborn screening program [15]. The quantitative test for G6PD activity of red blood cells was performed to confirm the diagnosis of G6PD deficiency in those who had positive results from screening.

### 2.4. Statistical Analyses

The statistics were compiled using a commercially available program (SPSS 12.0 for Windows, SPSS Inc., IL, USA). Categorical variables were analyzed using the chi-square, Fisher's exact, or Likelihood-ratio tests. Logistic regression was used to examine relationships among variables. Significance was defined as *P* < 0.05.

## 3. Results

A total of 1148 healthy infants at 25 to 45 days of age were enrolled into this study. Among them, 295 infants (25.7%) had TcB value ≥ 5 mg/dL. 

### 3.1. Demographic and Clinical Information

Table 1 compares the birth and clinical data of infants with TcB value ≥ 5 mg/dL to those with TcB value < 5 mg/dL. Late-preterm birth was more common in infants with TcB value ≥ 5 mg/dL than those without it (*P* = 0.024). In addition, there were significant differences in the feeding type and stool pattern between those infants with TcB value ≥ 5 mg/dL and those with TcB value < 5 mg/dL. TcB value ≥ 5 mg/dL was more common in infants with breast feeding (*P* < 0.001) and stool passage > 4 times per day (*P* < 0.001). The other characteristics—including gender, birth weight, G6PD deficiency, and weight gain—carried no significant difference between the two groups.

During the second visit at two months of age, no significant pathology associated jaundice was detected.

### 3.2. Risk Assessment

The risk assessment for prolonged jaundice (TcB value ≥ 5 mg/dL) by multivariate analysis is shown in Table 2. Gender, birth weight, gestational age, stool pattern, and feeding type were incorporated into the multivariate logistic regression model. Late-preterm infants were more likely to have TcB value ≥ 5 mg/dL than term infants (*P* = 0.032, OR = 1.998; 95% CI = 1.061–3.762). In addition, infants fed breast milk (*P* < 0.001, OR = 19.881; 95% CI = 8.243–47.95) and infants fed a combination of breast milk and formula (*P* = 0.001, OR = 4.539; 95% CI = 1.879–10.97) had a greater risk for TcB value ≥ 5 mg/dL than subjects fed formula.

**Figure 1:**
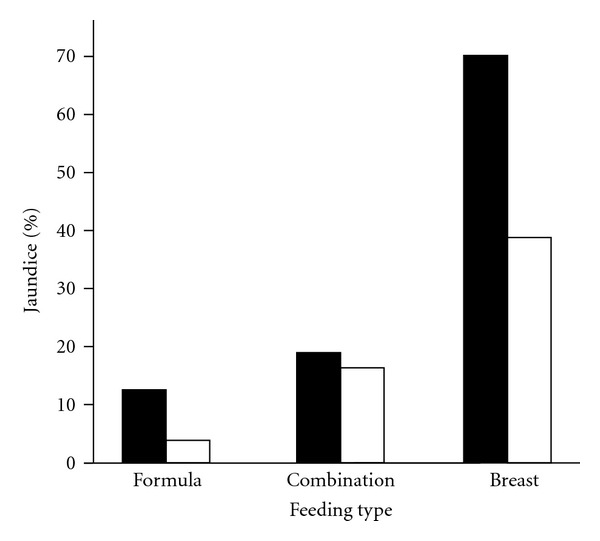
Incidence of jaundice, defined as TcB value ≥ 5 mg/dL, in infants at 25–45 days of age. Solid bar: gestational age from 34 to 36 weeks. Empty bar: gestational age from 37 to 42 weeks.

Figure 1 demonstrates the incidence of TcB value ≥ 5 mg/dL by feeding type and gestational age. The incidences of TcB value ≥ 5 mg/dL were 4.0% in formula-fed infants, 15.1% in combination-fed infants, and 39.8% in breast-fed infants (*P* < 0.001). Furthermore, TcB > 10 mg/dL was noticed in 1 infant who was fed formula (0.7%), 2 infants fed a combination of formula and breast milk (0.5%), and 54 infants fed breast milk (9.6%). In addition, jaundice was noted in 37.1% of preterm infants and 25.0% of term infants (*P* < 0.001).

### 3.3. Maternal Diet with Traditional Chinese Herbal Medicines

The association of traditional Chinese maternal diet with the parameters of nursing infants is shown in Table 3. TcB value ≥ 5 mg/dL was more common in breast-fed infants whose mothers did not consume a traditional Chinese postpartum diet than breast-fed infants whose mothers did consume such medicines and foods (*P* < 0.001). In addition, breast-fed infants whose mothers did not consume the traditional Chinese diet had more stool passage > 4 times per day than breast-fed infants whose mothers did consume the diet (*P* < 0.001). As for infants fed with formula or combination, there were no significant differences in the TcB levels and stool patterns between those with and those without the traditional Chinese postpartum diet. The other characteristics—including gestational age and weight gain—carried no significant difference.

## 4. Discussion

The current study depicts the prevalence of jaundice in otherwise healthy infants at one month of age. In this study, we used TcB as a surrogate for estimating bilirubin levels. TcB has been proven as effective as serum bilirubin in determining the extent of jaundice among term and late-preterm infants [16]. Because visible jaundice is approximately equal to a bilirubin value of 5 mg/dL, our study selected TcB value ≥ 5 mg/dL as an index of jaundice [17].

In Taiwan, consuming certain foods and Chinese medicines is widely believed to be beneficial for convalescing mothers during the postpartum period [13, 18]. However, the impact of maternal intake of a traditional Chinese postpartum diet on infants' health has never been surveyed [14, 19]. Our results indicate that a combination of Chinese medicines with a traditional Chinese maternal diet may decrease the development of prolonged jaundice. An increasing number of studies indicate that the content of breast milk is associated with breast milk jaundice [5–7]. Furthermore, changes in maternal diet can affect the composition of breast milk [20, 21]. These data imply that maternal diet is an important environmental factor for prolonged jaundice among breast-fed infants. Given the lack of association of maternal diet with prolonged jaundice in infants fed a combination of breast milk and formula in this study, we speculate that the effect of maternal diet is dose-dependent. A greater change in maternal diet would probably result in different rates of prolonged jaundice [20].

Some components of Chinese herbal medicines that have been proved as effective agents to enhance bilirubin clearance, such as *Glycyrrhiza uralensis*, are also consumed by mothers during their postpartum period [10, 11]. Therefore, the consumption of Chinese herbal medicines via mothers may affect the development of jaundice for breast-fed infants. In addition, in Taiwan it is a tradition that convalescing mothers ingest chicken soup flavored with alcohol. Chien et al. reported that the breast milk of these mothers contains significant levels of alcohol [14]. However, the impact of maternal alcohol consumption on prolonged jaundice of nursing infants is not clear. Further studies are needed to identify which components of Chinese herbal medicines or foods consumed by mothers are contributors to reduce the development of prolonged jaundice.

Our study presents the first finding to demonstrate a correlation of maternal diet with the stool pattern of breast-fed infants. There were documents reporting that breast milk jaundice is mediated by delay of bilirubin clearance from stool output [22, 23]. Our data may support the theory that enterohepatic circulation is accelerated, at least in part, by breast feeding.

This study identified breast feeding as the most important contributor for prolonged jaundice. The data are consistent with a large number of studies [24–26]. However, approximately 10% of breast-fed infants had a TcB value > 10 mg/dL in our study, which is higher than in previous reports with 2 to 4% of breast-fed infants at three weeks of age [25, 27]. This is probably due to variations in ethnicity and geography [8, 9]. In this study, another risk for prolonged jaundice was late-preterm birth. Jaundice was noticed in 70% of late-preterm infants fed breast milk. Furthermore, we found TcB values > 10 mg/dL in approximately 30% of late-preterm infants fed breast milk. As a result of immaturity in metabolism of bilirubin, late-preterm infants are at great risk of neonatal hyperbilirubinemia and prolonged jaundice. In addition, they are biologically vulnerable to bilirubin toxicity. These data lead to the conclusion that an aggressive approach to prolonged jaundice for early identification is of paramount important for preterm infants fed breast milk.

Some methodological issues should be cautiously interpreted in this study. First, pathological jaundice was not investigated [26, 28, 29]. But none of the infants in our study group had pathological jaundice upon followup at two months of age. Second, maternal diet was measured retrospectively by a self-reported questionnaire. Therefore, we cannot ascertain the cause effect of maternal diet on prolonged jaundice. Controlled trials would be necessary to clarify causal relationships. Third, we did not evaluate the long-term outcome of infants with prolonged jaundice. Further studies may be needed to evaluate the impact of prolonged jaundice on the neurological development of breast-fed infants, especially late preemies. 

## 5. Conclusion

This cohort study is the first to access the correlation of maternal diet with prolonged jaundice in nursing infants. The results suggest that maternal diet plays an important role in prolonged jaundice among breast-fed infants. Traditional Chinese dietary and herbal therapy may serve as an alternative management to prevent breast milk jaundice. We have depicted the clinical manifestations of prolonged jaundice among health and thriving infants and identified premature birth and breast feeding as contributors to prolonged jaundice. Our epidemiological data indicate prolonged jaundice is a common condition among breast-fed infants, especially when they are preterm. The results provide clinical implications for therapeutic strategies for prolonged jaundice. 

## Figures and Tables

**Table 1:** Correlation of birth and clinical information by TcB levels.

TcB value	<5 mg/dL (*n* = 853)	≥5 mg/dL (*n* = 295)	*P* value^∗^
Gender			0.172
Male	406 (47.6)
Female	447 (52.4)

Birth weight (g)			0.134
2000–2500	46 (5.4)
2501–4400	807 (94.6)

Gestational age (weeks)			0.024
34–36	44 (5.2)
37–42	809 (94.8)

G6PD status			0.757
Deficiency	20 (2.3)
Normal	833 (97.7)

Weight increase (g/d)			0.566
≤30	214 (25.1)
>30	639 (74.9)

Feeding milk			<0.001
Breast	338 (39.6)
Combination	370 (43.4)
Formula	145 (17.0)

Stool pattern (time/day)			<0.001
>4	248 (29.1)
2–4	254 (29.8)
<2	351 (41.1)

^∗^chi-square test.

**Table 2:** Risk assessment for prolonged jaundice by multivariate logistic regression analysis.

Characteristics	Adjusted OR	95% CI	*P* value
Gender (%)			
Male	1.294	0.964–1.736	0.086
Birth weight (g)			
2000–2500	1.667	0.876–3.172	0.120
Gestational age (weeks)			
34–36	1.998	1.061–3.762	0.032
Stool pattern (time/day)			
>4	1.187	0.809–1.742	0.382
2–4	1.168	0.790–1.726	0.437
Feeding milk			
Breast	19.881	8.243–47.95	<0.001
Combination	4.539	1.879–10.97	0.001

**Table 3:** Maternal diet with traditional Chinese herbal medicines during the postpartum period.

Feeding type	Formula			Combination			Breast		
Maternal diet with traditional Chinese herbal medicines	Yes (*n* = 31)	No (*n* = 120)	*P* value	Yes (*n* = 200)	No (*n* = 236)	*P* value	Yes (*n* = 266)	No (*n* = 295)	*P* value
TcB value (mg/dL) (%)			0.347^∗^			0.732			<0.001
<5	31 (100)	144 (95.0)		171 (85.5)
≥5	0 (0)	6 (5.0)		29 (14.5)

Gestational age (weeks) (%)			0.527^∗^			0.177			0.058
34–36	2 (6.5)	14 (11.7)		9 (4.5)
37–42	29 (93.5)	106 (88.3)		191 (95.5)

Stool pattern (time/day) (%)			0.707^†^			0.203			<0.001
>4	2 (6.5)	10 (8.3)		37 (18.5)
2–4	10 (32.2)	30 (25.0)		61 (30.5)
<2	19 (61.3)	80 (66.7)		102 (51.0)

Weight increase (g/d) (%)			0.260			0.122			0.796
5–30	10 (32.3)	27 (22.5)		56 (28.0)
>30	21 (67.7)	93 (77.5)		144 (72.0)

^∗^Fisher's exact test.

^†^Likelihood-ratio test.

## References

[1] Dennery PA, Seidman DS, Stevenson DK (2001). Drug therapy: neonatal hyperbilirubinemia. *The New England Journal of Medicine*.

[2] Mackinlay GA (1993). Jaundice persisting beyond 14 days after birth. *British Medical Journal*.

[3] Schneider AP (1986). Breast milk jaundice in the newborn. A real entity. *Journal of the American Medical Association*.

[4] Hargreaves T (1970). Breast-milk jaundice. *British Medical Journal*.

[5] Ince Z, Coban A, Peker I, Can G (1995). Breast milk *β*-glucuronidase and prolonged jaundice in the neonate. *Acta Paediatrica*.

[6] Uras N, Tonbul A, Karadag A, Dogan DG, Erel O, Tatli MM (2010). Prolonged jaundice in newborns is associated with low antioxidant capacity in breast milk. *Scandinavian Journal of Clinical and Laboratory Investigation*.

[7] Kumral A, Ozkan H, Duman N, Yesilirmak DC, Islekel H, Ozalp Y (2009). Breast milk jaundice correlates with high levels of epidermal growth factor. *Pediatric Research*.

[8] Chang PF, Lin YC, Liu K, Yeh SJ, Ni YH (2009). Prolonged unconjugated hyperbiliriubinemia in breast-fed male infants with a mutation of uridine diphosphate-glucuronosyl transferase. *Journal of Pediatrics*.

[9] Bozkaya OG, Kumral A, Yesilirmak DC (2010). Prolonged unconjugated hyperbilirubinaemia associated with the haem oxygenase-1 gene promoter polymorphism. *Acta Paediatrica*.

[10] Ho NK (1996). Traditional Chinese medicine and treatment of neonatal jaundice. *Singapore Medical Journal*.

[11] Fok TF (2001). Neonatal jaundice—traditional Chinese medicine approach. *Journal of Perinatology*.

[12] Huang W, Zhang J, Moore DD (2004). A traditional herbal medicine enhances bilirubin clearance by activating the nuclear receptor CAR. *Journal of Clinical Investigation*.

[13] Ho M, Li TC, Su SY (2011). The association between traditional Chinese dietary and herbal therapies and uterine involution in postpartum women. *Evidence-Based Complementary and Alternative Medicine*.

[14] Chien YC, Liu JF, Huang YJ, Hsu CS, Chao JCJ (2005). Alcohol levels in Chinese lactating mothers after consumption of alcoholic diet during postpartum “doing-the-month” ritual. *Alcohol*.

[15] Weng YH, Chou YH, Lien RI (2003). Hyperbilirubinemia in healthy neonates with glucose-6-phosphate dehydrogenase deficiency. *Early Human Development*.

[16] Rubaltelli FF, Gourley GR, Loskamp N (2001). Transcutaneous bilirubin measurement: a multicenter evaluation of a new device. *Pediatrics*.

[17] Kramer LI (1969). Advancement of dermal icterus in the jaundiced newborn. *American Journal of Diseases of Children*.

[18] Chuang CH, Chang PJ, Hsieh WS, Tsai YJ, Lin SJ, Chen PC (2009). Chinese herbal medicine use in Taiwan during pregnancy and the postpartum period: a population-based cohort study. *International Journal of Nursing Studies*.

[19] Liu-Chiang CY (1995). Postpartum worries: an exploration of Taiwanese primiparas who participate in the Chinese ritual of tso-yueh-tzu. *Maternal-Child Nursing Journal*.

[20] Uhari M, Alkku A, Nikkari T, Timonen E (1985). Neonatal jaundice and fatty acid composition of the maternal diet. *Acta Paediatrica Scandinavica*.

[21] Amato M, Berthet G, Von Muralt G (1988). Influence of fatty diet on neonatal jaundice in breast-fed infants. *Acta Paediatrica Japonica*.

[22] De Carvalho M, Robertson S, Klaus M (1985). Fecal bilirubin excretion and serum bilirubin concentrations in breast-fed and bottle-fed infants. *Journal of Pediatrics*.

[23] Alonso EM, Whitington PF, Whitington SH, Rivard WA, Given G (1991). Enterohepatic circulation of nonconjugated bilirubin in rats fed with human milk. *Journal of Pediatrics*.

[24] Gartner LM, Arias IM (1966). Studies of prolonged neonatal jaundice in the breast-fed infant. *Journal of Pediatrics*.

[25] Winfield CR, MacFaul R (1978). Clinical study of prolonged jaundice in breast- and bottle-fed babies. *Archives of Disease in Childhood*.

[26] Hannam S, McDonnell M, Rennie JM (2000). Investigation of prolonged neonatal jaundice. *Acta Paediatrica*.

[27] Clarkson JE, Cowan JO, Herbison GP (1984). Jaundice in full term healthy neonates—a population study. *Australian Paediatric Journal*.

[28] Rodie ME, Barclay A, Harry C, Simpson J (2011). NICE recommendations for the formal assessment of babies with prolonged jaundice: too much for well infants?. *Archives of Disease in Childhood*.

[29] Weng YH, Chiu YW (2009). Spectrum and outcome analysis of marked neonatal hyperbilirubinemia with blood group incompatibility. *Chang Gung Medical Journal*.

